# Thermal, Structural, and Molecular Characterization of a Model Cereal–Milk Infant Formula: Impact of Ultrasound Pre‐Treatment Prior to Spray Drying

**DOI:** 10.1002/fsn3.72043

**Published:** 2026-06-27

**Authors:** Yamen Barakat, Ilyas Atalar, Nevzat Konar

**Affiliations:** ^1^ Dairy Technology Department Ankara University Agriculture Faculty Ankara Turkiye; ^2^ Food Engineering Department Eskisehir Osmangazi University, Agriculture Faculty Eskisehir Turkey; ^3^ Ankara University, Institute of Food Safety Ankara Turkiye

**Keywords:** cereal‐milk matrix, glass transition temperature, infant formula, microstructure, spray drying, ultrasound‐pretreatment

## Abstract

This study investigated the impacts of an ultrasound (US) pre‐treatment applied to a liquid feed suspension prior to conventional spray drying to overcome the inherent stickiness and low yield challenges in a complex, carbohydrate‐rich model cereal–milk infant and toddler complementary food formulation. The effects of US pre‐treatment duration (30–90 s), spray dryer inlet temperature (150°C–180°C), and feed rate (7–12 mL/min) were evaluated using a full‐factorial response surface design. The findings demonstrated that acoustic cavitation applied as a feed pre‐treatment significantly reduced the viscosity of the suspension and improved subsequent atomization efficiency, which ultimately increased the powder recovery yield from approximately 12% to over 30%. Furthermore, the micro‐structural modifications and improved fat encapsulation induced during the ultrasound pre‐treatment step led to enhanced powder reconstitution properties, significantly increasing solubility. Microstructural and thermal evaluations revealed that the optimized processing parameters produced finer, less agglomerated particles that maintained an amorphous glassy state (T_g_ ranging from 17.5°C to 20.3°C) with preserved protein secondary structures. Notably, the accelerated drying kinetics resulting from the enhanced feed atomization limited the progression of the Maillard reaction, keeping advanced glycation products like HMF well within safe limits. Overall, this study highlights that an ultrasound pre‐treatment is a highly effective, non‐thermal feed‐modification strategy to optimize process efficiency, physical stability, and nutritional integrity in the spray drying production of complex multi‐component infant formulas.

## Graphical abstract

This study evaluated the efficacy of an ultrasound pre‐treatment applied to a complex cereal–milk infant formula prior to conventional spray drying to mitigate inherent stickiness and yield challenges. Acoustic cavitation (30–90 s) significantly reduced suspension viscosity and improved atomization efficiency, elevating powder recovery yield from 12% to over 30%. Microstructural modifications facilitated finer, less agglomerated particles with enhanced fat encapsulation and solubility (> 50%). Thermal analyses confirmed particles maintained an amorphous glassy state (Tg 17.5°C–20.3°C) with HMF levels within safe limits, demonstrating this non‐thermal strategy effectively optimizes process efficiency and nutritional integrity.

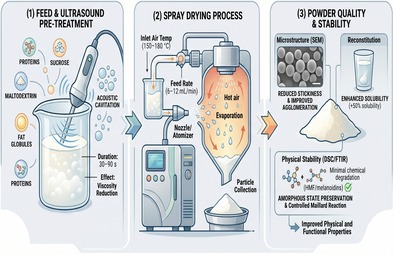

## 1 Introduction

Nutrition is the most critical environmental factor for growth, neurodevelopment, and the establishment of immunological defense mechanisms in the early stages of life. During this sensitive period, breast milk is considered the gold standard that fully meets the physiological requirements of infants with its bioactive components such as oligosaccharides (HMOs), lactoferrin, growth factors, and immunoglobulins, in addition to its macro‐ and micro‐nutrients (Luo et al. 2023; Soyyılmaz et al. 2021). Large‐scale epidemiological data indicate that breastfeeding significantly reduces infection‐related morbidity and mortality, while also providing long‐term protective effects against chronic metabolic diseases like obesity and diabetes (Victora et al. 2016; Noel et al. 2021). In this context, the World Health Organization (WHO) recommends exclusive breastfeeding for the first 6 months of life as a global public health strategy (Ekubay et al. 2018). However, when breastfeeding is not possible due to physiological insufficiencies, maternal medical conditions, or socio‐economic factors, infant formulas serve as alternatives designed to mimic the nutritional profile and functional properties of breast milk (Bridge 2021). Infant formulas are provided as alternatives or supplements to breastfeeding for at least the first 12 months, and their compositions are standardized to mimic the protein, carbohydrate, and lipid levels of human milk (Custodio‐Mendoza et al. 2024).

The industrial‐scale production of infant formula is a complex process aiming to ensure strict microbiological safety standards while preserving the stability of sensitive ingredients in the formulation (Ahmad and Guo 2021). To optimize storage, transportation, and shelf‐life stability, a powder form is generally preferred. In converting a liquid formulation into a powdered product, spray drying is widely used for its continuous operation, high production capacity, and relatively short processing time (Lin et al. 2018; Moejes and Van Boxtel 2017). Spray drying is based on the principle of drying the atomized fluid material within a hot air stream in a matter of seconds (Hanus and Langrish 2007). However, drying products containing a high proportion of lactose and low‐molecular‐weight sugar derivatives, such as infant formulas, presents significant technological challenges because these components possess relatively low glass transition temperatures (Tg) (Bhandari et al. 1997; Rannou et al. 2015). When the temperature in the drying environment exceeds the Tg of the particles, the product transitions from a glassy state to a rubbery state, increasing inter‐particle cohesion and leading to stickiness problems (Boonyai et al. 2004; Palzer 2005).

The stickiness issue is the most significant operational limitation, causing product accumulation on the inner walls of the drying chamber, cyclone blockages, and a significant decrease in process yield (Ozmen and Langrish 2003). When the product accumulates on the walls and remains in contact with the hot surface for a prolonged period, it undergoes thermal degradation, which can lead to scorched particles and quality losses. Previous studies suggest methods such as lowering the drying chamber wall temperature, using cold‐air sweeping systems, or adding carrier agents such as maltodextrin to the formulation (Jayasundera et al. 2011; Keshani et al. 2015). While the use of carrier agents in high proportions may limit the product formulation's nutritional content, lowering the wall temperature alters the enthalpy of the drying air, potentially leading to insufficient particle drying and an increase in moisture content (Lin et al. 2018). Therefore, to solve the stickiness problem and yield losses, it is necessary to optimize not only the drying parameters but also the physicochemical properties of the feed liquid.

In addition to physical problems, the high inlet air temperatures (typically 150°C–200°C) applied during spray drying can trigger reactions that threaten the chemical and nutritional quality of infant formulas (Carter et al. 2018). Infant formula matrices, which are particularly rich in proteins and reducing sugars (lactose, glucose), provide an ideal substrate for Maillard reactions (Jiang and Guo 2021). While the blockage of essential amino acids such as lysine during thermal processing leads to a loss of nutritional value, potentially toxic compounds like 5‐hydroxymethylfurfural (HMF) and advanced glycation end products (AGEs) can form in the later stages of the reaction (Hendricks and Guo 2014; Custodio‐Mendoza et al. 2024). Considering the still‐developing detoxification mechanisms of infants, keeping HMF levels in infant formulas below legal limits and minimizing thermal damage are critical for food safety. Furthermore, high temperatures can cause the oxidation of polyunsaturated fatty acids (PUFAs) in the formulation and the loss of heat‐sensitive bioactive components such as vitamin C (Rannou et al. 2015).

These limitations in current industrial applications have led researchers to integrate innovative, non‐thermal technologies to improve process efficiency while preserving product quality (Moejes and Van Boxtel 2017). The integration of innovative assisting technologies, such as ultrasonication or infrared radiation, prior to or during conventional thermal dehydration has been proven to significantly accelerate moisture diffusion kinetics and shorten total processing times, thereby safeguarding heat‐sensitive constituents from prolonged thermal degradation (Dehghan‐Manshadi et al. 2020). In this context, ultrasonication (US) technology stands out as a significant pre‐treatment alternative with its potential to modify the rheological properties, particle size, and emulsion stability of liquid food systems. Ultrasonication is based on the principle of the sudden formation and violent collapse of micro‐bubbles through the acoustic cavitation phenomenon it creates in the liquid medium (Chemat and Khan 2011). This physical event can alter the structure of the food matrix at a microscopic level by generating sudden pressure changes up to 1000 bar, local temperature increases reaching 5000 K, and high shear forces.

The integration of ultrasound pretreatment prior to spray drying relies on distinct physical mechanisms that must be critically evaluated as one moves from simplified models to multi‐component systems. In single‐component dairy streams, prior research has established clear benchmarks; for instance, Song et al. (2022) demonstrated that ultrasonication of isolated milk proteins reduced feed viscosity by 30%, thereby directly mitigating wall deposition from 19% to 14% by generating finer droplets during atomization. Similarly, Sun et al. (2018) observed that acoustic cavitation partially unfolds protein secondary and tertiary structures, exposing interior hydrophobic groups and consequently improving fat globule encapsulation and emulsion stability. However, a critical comparison reveals that these established mechanisms cannot be directly extrapolated to composite food matrices without considering competitive interactions. While smaller droplet sizes inherently accelerate drying rates by expanding the heat‐ and mass‐transfer surface area (Schutyser et al. 2019), the presence of gelatinized starches and low‐molecular‐weight sugars, such as lactose and sucrose, drastically alters the surface phase transitions. In simplified protein models, viscosity reduction directly translates to superior flowability and lower stickiness. Conversely, in a complex cereal–milk matrix, the exposed hydrophobic groups of unfolded proteins must compete with the hydrophilic domains of disordered starches and highly hygroscopic sugars for water binding and surface orientation. Therefore, rather than merely summarizing isolated benefits, this study critically contrasts how these competing molecular phenomena modulate macroscopic powder characteristics, addressing a clear gap where individual processing advantages often conflict in composite infant food formulations. Furthermore, in complex cereal–milk composite matrices, dynamic state transitions extend beyond simple stickiness to localized structural collapse and crystalline rearrangements under subsequent thermal stress. Specifically, the sonication‐induced exposure of hydrophobic protein domains can paradoxically accelerate the formation of amorphous bridges if localized wetting occurs, as the modified surface energy influences the glass transition kinetics of lactose and other sugars during dehydration (Ozuna et al. 2014; Zhao et al. 2026). Moreover, modification of protein structures by acoustic cavitation may influence the stabilization of the protein‐lactose matrix, as shifts in secondary structure can alter the glass transition temperature and susceptibility to thermal events (Zhao et al. 2025). This structural reorganization, driven by high‐intensity cavitation, likely modulates the intensity of Maillard reaction pathways by altering the accessibility of reactive amino groups to reducing sugars (Gao et al. 2024).

When atomization or drying kinetics are insufficient, the final powder retains elevated residual moisture. Within an amorphous lactose‐sucrose network, even trace amounts of trapped water act as a potent plasticizer, drastically lowering the matrix's Tg and increasing molecular mobility at ambient temperatures. This state of increased mobility promotes time‐dependent crystallization and the subsequent collapse of the glassy matrix, which directly precipitates caking and stickiness issues in stored dairy powders (Roos 2002; Torres et al. 2017). Consequently, the uncontrolled release of moisture during this phase transition further facilitates the formation of liquid bridges between particles, creating a feedback loop that compromises the physical stability and structural integrity of the final product (Qi et al. 2023; Silalai and Roos 2015). Given these stability challenges, optimizing the balance between ultrasonic intensity and drying parameters remains essential to mitigate the adverse plasticizing effects of residual moisture and ensure long‐term shelf‐life (Chuy and Labuza 1994; Saxena et al. 2021).

The existing literature on ultrasound pretreatment prior to spray drying predominantly focuses on relatively simple, single‐ or bi‐component systems, such as isolated milk proteins, fruit juices, or basic oil‐in‐water emulsions (Tong et al. 2022). A significant research gap remains regarding how acoustic cavitation alters the multi‐faceted phase interactions within highly complex, multi‐component matrices. Infant and toddler complementary food formulations represent intricate colloidal systems where milk proteins, complex carbohydrates (starch), low‐molecular‐weight sugars (lactose, sucrose), and lipids coexist and interact dynamically. The specific knowledge gap addressed in this study is the lack of understanding of how ultrasound pre‐treatment simultaneously modulates matrix‐specific phenomena, such as starch short‐range molecular reordering, protein‐lipid encapsulation dynamics, and specific Maillard reaction kinetics, under subsequent thermal processing stress. While recent reviews have highlighted the potential of non‐thermal technologies in cereal‐based infant foods (Pasdar et al. 2024), systematic research explicitly linking ultrasound‐induced structural modifications to macroscopic drying characteristics, stickiness dynamics, and the glass transition temperature (Tg) is virtually nonexistent. To fill this gap, this study novelly applies a holistic approach to a model cereal–milk infant formulation, explicitly investigating how the physical effects of cavitation can mitigate industrial spray drying limitations like wall deposition and low yield while safeguarding the physical and chemical integrity of a highly composite food matrix.

This study aims to address the effects of ultrasonication (US) pretreatment applied prior to spray drying on the production of model infant and toddler complementary food formulations using a holistic approach. The primary hypothesis of the study is that the physical forces generated by the ultrasonic pre‐treatment will modify the rheological properties of the complex formula suspension, enhance atomization efficiency, and strengthen emulsion stability, thereby maximizing spray‐drying recovery. Furthermore, it is hypothesized that the structural modifications and reduced viscosity will indirectly influence the thermal degradation profile of the powders. Specifically, by enabling the formation of finer droplets and accelerating the evaporation kinetics, the pre‐treatment is proposed to shorten the critical residence time during which particles retain high moisture content under elevated temperatures. Consequently, this rapid transition into a glassy state with restricted molecular mobility may kinetically limit the progression of the Maillard reaction, thereby potentially mitigating the accumulation of advanced glycation end‐products like HMF, counteracting the initial localized thermal energy input of acoustic cavitation.

## 2 Materials and Methods

### 2.1 Materials

Whole milk powder, rice flour, wheat flour, wheat starch, semolina, sucrose, oat flour, vitamin C, sodium chloride, vanilla, lecithin, and sodium bicarbonate used in the preparation of the infant formula samples were obtained from the local market in Ankara. All chemicals used in the analytical studies were of analytical grade.

### 2.2 Study Design and Sample Preparation

The levels of the independent variables were determined through preliminary screening experiments to accommodate the physical limitations of the laboratory‐scale equipment and the thermal sensitivity of the formulation. Specifically, the spray dryer inlet temperature was set between 150°C and 180°C; preliminary tests indicated that temperatures below 150°C resulted in incomplete evaporation and chamber wall wetting, whereas temperatures exceeding 180°C caused severe thermal degradation (browning) and instantaneous caking of the sucrose‐rich matrix. The feed rate was constrained to 7–12 mL/min to closely match the aspirator's evaporative capacity, ensuring stable nozzle atomization without dripping. Lastly, ultrasonication pre‐treatment was evaluated at 30, 60, and 90 s; treatments shorter than 30 s provided insufficient acoustic cavitation for a measurable reduction in suspension viscosity, while intervals beyond 90 s generated excessive localized heating that could prematurely denature the milk proteins prior to atomization. The experimental plan was structured using a full‐factorial Response Surface Methodology (RSM) design (*n* = 12).

For the infant formula powder mixture, a solid blend consisting of whole milk powder (19.5 g/100 g), rice flour (19.5 g/100 g), wheat flour (25.0 g/100 g), wheat starch (3.80 g/100 g), semolina (3.00 g/100 g), sucrose (27.31 g/100 g), oat flour (1 g/100 g), vitamin C (0.04 g/100 g), sodium chloride (0.15 g/100 g), vanilla (0.10 g/100 g), sunflower lecithin (0.50 g/100 g), and sodium bicarbonate (0.10 g/100 g) was mixed using a high‐speed blender until a homogeneous structure was achieved. Subsequently, this mixture was diluted with distilled water to obtain a total water‐soluble dry matter content of 10%–15% and pasteurized at 85°C for 5 min using a thermal mixer. Certain structural limitations must be acknowledged when extrapolating these laboratory‐scale trends to commercial infant formula processing lines. Commercial formulations typically undergo rigorous micronutrient fortification, incorporating complex mineral pre‐mixes (e.g., calcium phosphates, iron sulfates) and precise blends of vegetable or marine oils rich in polyunsaturated fatty acids (PUFAs). The restriction of the lipid phase to inherent milk fat and lecithin in this model system means that potential interactions between sound‐wave shear fields and highly susceptible double bonds of long‐chain PUFAs, which could alter localized lipid peroxidation kinetics, were not completely captured. Furthermore, the absence of isolated, highly heat‐sensitive bioactive fractions (such as lactoferrin or immunoglobulins) restricts the direct extrapolation of these non‐thermal feed modifications regarding absolute bioactive preservation.

The ultrasonication pre‐treatment was performed using an ultrasonic probe system (Bandelin HD 4400, 20 kHz, 1000 W, Germany). To address the potential for localized thermal degradation induced by acoustic cavitation, the process was conducted under strictly isothermal conditions. The treatment chamber was integrated with a double‐jacketed cooling system, coupled with a circulating water bath maintained at 10.0°C ± 1.0°C, which effectively dissipated heat and maintained a stable suspension temperature throughout the process. For the 30, 60, and 90 s treatments, the total energy inputs recorded by the ultrasonic processor were approximately 8.4–8.7 kJ, 16.1–18.3 kJ, and 26.1–27.5 kJ, respectively. Continuous monitoring confirmed that no significant temperature rise occurred during the 30–90 s treatment intervals, supporting the classification of this ultrasound application as a non‐thermal pre‐treatment strategy. A non‐ultrasonicated suspension was used as the control group.

The ultrasonicated feed suspensions were converted into powder using a laboratory‐scale spray dryer (Unopex, İzmir, Türkiye). The device was equipped with a dehumidifier unit and an inert loop system. The process parameters kept constant during drying were aspirator capacity at 90% (approximately 35 m^3^/h air flow), atomization air flow at 600 L/h (40 mm), and a two‐fluid nozzle type with a 1.0 mm diameter. The independent variables, feed rate (7 and 12 mL/min) and inlet air temperature (150°C and 180°C), were applied according to the experimental design. During steady‐state spray dryer operation, the outlet air temperature was continuously monitored and recorded as a dependent process indicator. The outlet temperature stabilized within the range of 68°C to 76°C for the drying runs conducted at an inlet air temperature of 150°C, and ranged between 82°C and 94°C for the runs processed at an inlet air temperature of 180°C, directly correlating with the dynamic moisture load entering the system. Following the drying process, the samples were stored in airtight and moisture‐proof polyethylene bags at 4.00°C ± 1.00°C until subsequent analysis.

### 2.3 Analysis

#### 2.3.1 Physico‐Chemical Analysis

The water activity of the infant formula samples was determined at 20°C using a water activity meter (Aqualab 4TE, Munich, Germany). Moisture content was determined using a vacuum oven (approximately 2.00 g sample, 100°C–102°C). pH analyses were conducted using a pH meter (Testo 206, Titisee‐Neustadt, Germany) on samples prepared by dissolving 1.00 g of powder in 9 mL of deionized water with the aid of a magnetic stirrer. A 3‐point calibration (pH 4, 7, and 10) was performed using standard buffer solutions prior to the measurements.

For ash analysis, porcelain crucibles were initially heated in an oven at 150°C for 2 h to remove moisture, then placed in a desiccator for at least 1 h to reach a constant weight at room temperature. After recording the tare weight of the numbered crucibles at constant weight (M1), approximately 3 g of homogeneous sample was added to the crucibles (m1). The samples were incinerated in a furnace (CLMF‐210, CLS Scientific, Ankara, Turkey) set at 550°C for 6 h. Once the furnace temperature dropped below 300°C, the samples were transferred to a desiccator and allowed to cool to room temperature. The final mass of the crucibles (M2) was weighed on an analytical balance, and total ash contents were calculated using Equations (1) and (2):

(1) $$\mathrm{Ash}\;\text{content}\;\left(\%\right)=\left[\left(M2-M1\right)/\text{m1}\right]\times 100$$

(2) $$\mathrm{Ash}\;\text{content in}\;\mathrm{dry}\;\text{matter}=\left(\mathrm{Ash}\;\text{content}\;\left(\%\right)/\mathrm{Dry}\;\text{matter}\;\left(\%\right)\right)\times 100$$

#### 2.3.2 Particle Size Distribution

The particle size distribution properties of the powder samples were determined using a laser diffraction particle size analyzer (Mastersizer 3000, Malvern, Cambridge, UK). For this aim, 2.00 g of the sample was dispersed in distilled vegetable oil and placed in the sample chamber for readings. The analysis yielded the median (D3/2, μm) and volume‐weighted mean particle size (D4/3, μm), as well as the D10, D50, and D90 (μm) values and specific surface areas of the samples.

#### 2.3.3 Color and Browning Index (BI)

Color parameters (L*, a*, and b*) were determined using a colorimeter (CR400, Konica Minolta, Kyoto, Japan) according to a colorimetric method. In this system, L* represents lightness, a* denotes green (−a*) or red (+a*) hues, and b* indicates blue (−b*) or yellow (+b*) hues. The browning index (BI) of the samples was calculated using Equation (3) and (4) (Martinez‐Alvarenga et al. 2014).

(3) $$X=\left(a^{*}+1.75L^{*}\right)/\left(5.64{\text{5L}}^{*}+a^{*}-b^{*}\right)$$

(4) $$\mathrm{BI}=\left[100\;\left(X-0.31\right)\right]/0.172$$

where BI; browning index. X value is dimensionless.

#### 2.3.4 Drying Efficiency

To determine the drying efficiency, Equation (5) was used (Nunes and Mercadante 2007).

(5) $$\mathrm{DY}\;\left(\%\right)=\left(\mathrm{DC}/\mathrm{DG}\right)\times 100$$

where DG is the total mass before drying (g), and DC is the total mass obtained by the drying process (g).

#### 2.3.5 Wettability and Solubility

To determine the wettability properties of the powders, 5 g of powder was weighed, and the time required for it to completely submerge in 100 mL of distilled water through a calibrated funnel (50 mm width) was recorded (Barkouti et al. 2013). To determine water solubility, 2.5 g of the powder sample was weighed into 30 mL of distilled water and incubated at 37°C for 30 min. Following incubation, the mixture was centrifuged at 3500 rpm and 4°C for 20 min. The resulting supernatant was collected and dried at 105°C until a constant weight was achieved (Demirci et al. 2025).

(6) $$\begin{array}{c} \text{Water Solubility Index}\;\left(\%\right)=\\ \left(\text{Dried supernatant}/\text{Initial sample weight}\right)\times 100 \end{array}$$

#### 2.3.6 Bulk and Tapped Densities

Bulk density is defined as the mass per unit volume. Tapped density is measured by determining the volume occupied by a specific amount of powder after a specified number of taps. Graduated cylinders of 10 mL were used for the measurements. The loose bulk density (ρL) was determined by dividing the mass of the powder by its read volume after being placed in the cylinder. The tapped density (ρT) was determined by dividing the mass by the final read volume after 125 manual taps.

#### 2.3.7 Flowability (CI) and Cohesiveness (HR)

Flowability (Carr Index, CI) and cohesiveness values (Hausner Ratio, HR) were calculated using the loose (ρL) and tapped (ρT) bulk density results and the following Equation (7) and (8):

(7) $$\mathrm{CI}=100\times \left(\mathrm{\rho T}-\mathrm{\rho L}\right)/\mathrm{\rho L}$$

(8) HR=ρT/ρL

#### 2.3.8 Hygroscopicity

The method developed by Fritzen‐Freire et al. (2012) was used to determine the hygroscopic characteristics of the samples. A 1 g sample was placed in airtight containers at 25°C alongside a saturated salt solution, and the amount of moisture adsorbed after 1 week was determined. Hygroscopicity was expressed as the amount of moisture adsorbed (g) per 100 g of dry matter.

#### 2.3.9 Amadori Compounds and Melanoidin Contents

The Maillard reaction consists of three stages: initial, intermediate, and advanced. Colorless Amadori compounds form in the initial stage and can be detected spectrophotometrically at 304 nm. As the reaction progresses to the intermediate and advanced stages, brown polymers called melanoidins form, which can be detected at 420 nm. The formation of Amadori compounds and melanoidins during conjugation was determined as described by Zha et al. (2019). The dried powders were dissolved in distilled water to obtain a 1 mg/mL solution, and the absorbances were measured at 304 and 420 nm using a UV–Vis spectrometer (UV‐1280, Shimadzu, Kyoto, Japan) to determine the formation of Amadori compounds and melanoidins, respectively.

#### 2.3.10 Determination of Secondary Structure Changes

The FTIR spectra of the infant formula powders were determined using a diamond triple‐bounce ATR accessory (Bruker Alpha, Germany) in the mid‐infrared range (4000–400 cm^−1^) at a resolution of 4 cm^−1^ with 32 scans.

#### 2.3.11 HMF Content

The sample preparation step was performed according to Bogdanov (2009). A 5 g sample was weighed and dissolved in 25 mL of distilled water. Then, 0.5 mL of Carrez‐I (15%, w/v, potassium hexacyanoferrate) and Carrez‐II (30%, w/v, zinc acetate dihydrate) solutions were added, and the final volume was adjusted to 50 mL. The supernatant obtained by centrifugation at 5000 rpm for 15 min at 4°C was analyzed for HMF according to Zappalà et al. (2005). A 5 mL aliquot of the supernatant was mixed with 10% (w/v) p‐toluidine solution, followed by the addition of 1 mL of 0.5% (w/v) barbituric acid. After 3 min, the absorbance was read at 550 nm against a blank sample.

#### 2.3.12 Differential Scanning Calorimetry (DSC) Analysis

Calorimetric measurements were performed with a TA Q20 model Differential Scanning Calorimeter (DSC) (TA Instruments, Newcastle, DE, USA). The DSC analyses were conducted on samples sealed in aluminum pans containing 20 μL. Nitrogen was used as the carrier gas in all DSC studies, with a flow rate of 50 mL/min (Tsai et al. 2018). The thermal profile was initiated by cooling the samples to −20°C, followed by a controlled heating scan from −20°C to 120°C at a constant heating rate of 10°C/min. An empty aluminum pan was used as a reference. The glass transition temperature (Tg) was calculated using the midpoint method, based on the inflectional baseline step change associated with the second‐order thermodynamic transition.

#### 2.3.13 Scanning Electron Microscopy (SEM)

An SEM device was used to examine the internal and external microstructural properties of the samples. Powder samples were placed on SEM sample holders using double‐sided adhesive tape. To examine the internal structure, a second adhesive tape was applied over the powders affixed to the holder, and the tape was then pulled rapidly to fracture the particles. The weakness of this sectioning method is that only fragile particles, especially hollow ones, can be fractured. Therefore, a strong adhesive tape was used for each sample. All samples were subsequently coated with Pt–Pd using a Model MSP‐1S magnetron sputter coater manufactured by Vacuum Device Inc. (Tokyo, Japan). The coated samples were analyzed using an SEM operating at 15 kV (Soottitantawat et al. 2005).

#### 2.3.14 Statistical Analysis

The statistical evaluation of the experimental data was performed using the Design‐Expert 13.0 software package (Stat‐Ease Inc., Minneapolis, MN, USA). A full‐factorial Response Surface Methodology (RSM) was employed to determine the main and interaction effects of the independent variables on the measured responses. Analysis of Variance (ANOVA) was utilized to assess the significance of the developed polynomial regression models and individual terms. The adequacy and quality of the fitted models were rigorously evaluated using the coefficient of determination (*R* ^2^), adjusted *R* ^2^, predicted *R* ^2^, and adequate precision (signal‐to‐noise ratio). Furthermore, two‐way interaction effects between the factors (e.g., temperature × ultrasonication time) were quantified to understand synergistic or antagonistic behaviors during processing. Model terms and interactions with a significance value (*p*) less than 0.05 were considered statistically significant, adhering to the standard significance notation (* for *p* < 0.05, ** for *p* < 0.01, and *** for *p* < 0.001) throughout the manuscript.

## 3 Results and Discussion

### 3.1 Yield and Physico‐Chemical Properties

The mathematical model established for the powder recovery yield was statistically significant (*p* < 0.05). To systematically separate the independent impacts of the feed pre‐treatment from the subsequent spray drying configurations, the main and interaction effects were rigorously partitioned via ANOVA (Table 1). The statistical analysis revealed that the process yield was not governed by a single generalized factor; instead, it was independently and significantly (*p* < 0.05) determined by all three main effects: Inlet Air Temperature (A), Feed Rate (B), and Ultrasonication Pre‐treatment Time (C). Crucially, a significant two‐way interaction effect between Feed Rate and Ultrasonication Time (B × C) was established (*p* < 0.05), providing robust empirical evidence that the physical benefits of acoustic cavitation on powder recovery are deeply interconnected with the mass‐transfer load entering the drying system. These baseline yield values, while seemingly low, are consistent with the literature on laboratory‐scale spray dryers, where small drying chamber diameters and high surface‐area‐to‐volume ratios inherently lead to higher wall deposition and lower cyclone recovery efficiency than in industrial‐scale configurations. This mechanism aligns with the findings of Younesi et al. (2023), who reported that structural modifications of WPC via ultrasonication drastically lower feed viscosity, preferentially driving surface‐active proteins to the particle interface to form a non‐adherent film that mitigates chamber‐wall stickiness.

**TABLE 1:** General factorial experimental design with three independent variables and their effects on drying yield, moisture content, and water activity of spray‐dried samples.

Sample	A (°C)	B (mL/min)	C (s)	Yield (%)	Moisture Content (g/100 g)	Water Activity	Ash Content (g/100 g)	pH	Protein Content (g/100 g)
1	150	7	30	12.15 ± 1.50	1.95 ± 0.21	0.1462 ± 0.021	1.48 ± 0.27	6.92 ± 0.33	8.99 ± 0.07
2	180	7	30	16.26 ± 1.90	1.48 ± 0.45	0.1340 ± 0.029	1.49 ± 0.19	7.06 ± 0.67	8.93 ± 0.12
3	150	12	30	13.28 ± 1.74	2.01 ± 0.36	0.1934 ± 0.038	1.50 ± 0.33	6.92 ± 0.14	8.66 ± 0.15
4	180	12	30	13.76 ± 1.65	1.71 ± 0.42	0.1792 ± 0.034	1.49 ± 0.27	7.04 ± 0.29	8.68 ± 0.13
5	150	7	60	26.98 ± 1.49	1.86 ± 0.38	0.1323 ± 0.029	1.46 ± 0.18	6.84 ± 0.13	8.95 ± 0.09
6	180	7	60	31.27 ± 1.38	1.46 ± 0.19	0.1151 ± 0.011	1.43 ± 0.22	7.03 ± 0.19	8.61 ± 0.11
7	150	12	60	16.89 ± 1.17	2.73 ± 0.75	0.1759 ± 0.049	1.41 ± 0.31	7.06 ± 0.74	8.70 ± 0.05
8	180	12	60	20.15 ± 1.16	2.11 ± 0.37	0.1387 ± 0.027	1.45 ± 0.40	7.01 ± 0.61	8.92 ± 0.10
9	150	7	90	25.15 ± 1.75	1.72 ± 0.42	0.1287 ± 0.013	1.51 ± 0.46	6.95 ± 0.53	8.92 ± 0.04
10	180	7	90	31.24 ± 1.54	1.48 ± 0.22	0.1113 ± 0.011	1.46 ± 0.38	7.07 ± 0.91	9.03 ± 0.07
11	150	12	90	16.77 ± 1.16	2.46 ± 0.31	0.1640 ± 0.037	1.53 ± 0.28	7.01 ± 0.64	8.82 ± 0.08
12	180	12	90	22.80 ± 1.48	1.97 ± 0.29	0.1133 ± 0.035	1.49 ± 0.36	6.92 ± 0.71	8.47 ± 0.05
Model				Factorial	Factorial	Factorial	Factorial	Factorial	Factorial
Model *p*‐value				0.0149*	0.0010**	0.0605^ns^	0.317 ^ns^	0.4352^ns^	0.8804^ns^
Significant terms				A, B, C, B × C	A, B, C, B × C	A, B, C	nd	nd	nd
*R* ^2^				0.9967	0.9719	0.9862	0.9186	0.8808	0.6238
Adjusted *R* ^2^				0.9817	0.9381	0.9242	0.5523	0.3443	−1.0603
Predicted *R* ^2^				0.8803	0.8379	0.5040	−1.9302	−3.2921	−12.5442
Adeq Precision				22.50	16.69	12.90	5.02	4.28	1.9320
C.V. (%)				4.49	5.09	5.28	1.55	0.85	2.90
Standard deviation				0.9230	0.0973	0.0076	0.0229	0.0597	0.2551

*Note:* Values are expressed as mean ± standard deviation. All analyses were performed at least in triplicate. The experimental design consisted of a general factorial design with 12 experimental runs. Independent variables were spray dryer inlet temperature (150°C–180°C), feed rate (7–12 mL/min), and ultrasonication time (30–90 s). Goodness‐of‐fit measures for each response are listed at the bottom of the table.

Abbreviations: A, spray dryer inlet temperature; B, feed rate; C, ultrasonication time; nd, not determined.

* Indicates a significant model at *p* < 0.05.

** Indicates a significant model at *p* < 0.01; ns: non‐significant (*p* ≥ 0.05).

Increasing the ultrasonication time substantially improved the yield. For instance, while the yield was 12.15% with 30 s of ultrasound application at 150°C and 7 mL/min, it increased to 26.98% with 60 s and reached 25.15% with 90 s. Although direct rheological measurements of the feed suspension viscosity and pre‐drying droplet sizes were not conducted in this study, this physical improvement is quantitatively supported by the particle size distribution data of the final powders (Table 2). The Sauter mean diameter (D_3,2_), which is highly dependent on the initial atomized droplet size, was significantly reduced (*p* < 0.05) with prolonged ultrasonication. This measurable reduction in D_3,2_ serves as a robust quantitative indicator that acoustic cavitation effectively lowered the complex matrix's resistance to atomization. Consequently, the formation of finer droplets accelerated the evaporation rate, allowing the particles to transition into a non‐sticky glassy state before colliding with the dryer walls, thereby significantly increasing the powder collection yield.

**TABLE 2:** General factorial experimental design with three independent variables and their effects on solubility, wettability, hygroscopicity, particle size distribution parameters (D_4,3_ and D_3,2_), and specific surface area (SSA) of spray‐dried samples.

Run	A (°C)	B (mL/min)	C (s)	Solubility (%)	Wettability (min)	Hygroscopicity (g/100 g DM)	D_4,3_ (μm)	D_3,2_ (μm)	SSA
1	150	7	30	32.2 ± 0.17	30 ± 1	16.2 ± 0.11	6.97 ± 0.17	17.7 ± 1.05	860.8 ± 10.2
2	180	7	30	31.2 ± 0.15	31 ± 1	16.4 ± 0.13	7.34 ± 0.71	19.9 ± 1.25	817.5 ± 5.20
3	150	12	30	34.4 ± 0.51	29 ± 2	35.3 ± 0.12	7.19 ± 0.52	18.4 ± 1.03	834.8 ± 11.6
4	180	12	30	40.6 ± 0.67	27 ± 1	15.0 ± 0.16	7.93 ± 0.35	20.3 ± 2.04	756.2 ± 8.90
5	150	7	60	36.6 ± 0.11	33 ± 2	16.9 ± 0.12	6.85 ± 0.15	18.9 ± 0.90	912.2 ± 8.50
6	180	7	60	49.7 ± 0.69	28 ± 2	16.7 ± 0.17	6.24 ± 0.11	17.5 ± 0.75	961.7 ± 12.0
7	150	12	60	42.1 ± 1.28	24 ± 1	16.0 ± 0.14	7.29 ± 0.53	18.6 ± 1.20	823.4 ± 14.0
8	180	12	60	48.0 ± 0.19	25 ± 2	16.3 ± 0.13	6.99 ± 0.25	19.7 ± 0.45	858.4 ± 5.50
9	150	7	90	45.1 ± 1.30	27 ± 2	17.6 ± 0.12	6.38 ± 0.35	18.1 ± 0.93	939.7 ± 10.1
10	180	7	90	49.8 ± 1.50	23 ± 1	17.4 ± 0.16	6.48 ± 0.42	19.1 ± 0.89	926.1 ± 9.80
11	150	12	90	47.4 ± 0.40	20 ± 2	16.3 ± 0.14	7.16 ± 0.21	18.3 ± 0.74	837.7 ± 8.80
12	180	12	90	52.3 ± 0.18	17 ± 1	17.1 ± 0.15	7.10 ± 0.59	19.2 ± 0.65	854.1 ± 5.70
Model *p*‐value				0.0058**	0.0086**	0.6869^ns^	0.0768^ns^	0.5079^ns^	0.0633^ns^
Significant terms				A, C	B, C	nd	B, C	nd	B, C
*R* ^2^				0.9424	0.9321	0.7725	0.9824	0.8542	09856
Adjusted *R* ^2^				0.8732	0.8506	−0.2510	0.9031	0.1982	08206
Predicted *R* ^2^				0.6680	0.6089	−7.1886	0.3660	−4.2478	04804
Adeq Precision				10.52	10.25	3.38	12.1284	3.6467	12.8955
C.V. (%)				6.20	6.84	33.87	2.08	4.15	1.93
Standard deviation				2.63	1.79	6.12	0.1451	0.7810	16.68

*Note:* Values are expressed as mean ± standard deviation. All analyses were performed at least in triplicate. The experimental design consisted of a general factorial design with 12 experimental runs. Independent variables were spray dryer inlet temperature (150°C–180°C), feed rate (7–12 mL/min), and ultrasonication time (30–90 s). Goodness‐of‐fit measures for each response are listed at the bottom of the table.

Abbreviations: A, Spray dryer inlet temperature (°C); B, feed rate (mL/min); C, ultrasonication time (s); nd, not determined; SSA, Specific surface area; Tg, Glass transtion temperature.

** Indicates a significant model at *p* < 0.01; ns: non‐significant (*p* ≥ 0.05).

Elevating the inlet temperature from 150°C to 180°C generally enhanced the yield. The high temperature accelerated heat and mass transfer, allowing the surfaces of the particles to dry (crust formation) before colliding with the wall, thereby reducing stickiness. Interestingly, ultrasound (US) pretreatment for up to 90 s did not cause a further decline in yield. It is hypothesized that US‐induced viscosity reduction in the feed solution improved atomization quality, potentially compensating for the stickiness issues by creating more uniform droplet distributions, thereby preventing excessive nozzle clogging. Also, notably, the 60 s application approximately doubled the yield compared to the 30 s treatment. These results are consistent with the findings previously reported by Song et al. (2022), who stated that ultrasound reduces viscosity and enhances atomization efficiency.

Upon examining the models for moisture content (*p* < 0.05) and water activity (*p* > 0.05), it was observed that Temperature (A), Feed Rate (B), and Ultrasonication Time (C) influenced these parameters. As predicted, an increase in temperature reduced both the moisture content and water activity. For example, while the moisture content in Run 1 (150°C) was 1.95 g/100 g, it decreased to 1.48 g/100 g in Run 2 (180°C). Prolonging the ultrasonication time also contributed to the reduction in moisture content. The moisture content, which was 1.95 g/100 g for the 30 s application, dropped to 1.72 g/100 g for the 90 s application. This suggests that the microstructural changes and the smaller particle size induced by ultrasonication facilitated the diffusion of water from the particle interior to the surface and its subsequent evaporation. The physical modifications induced by power ultrasound accelerate moisture migration, thereby contributing to a significant reduction in water activity and the subsequent microbial load (Sakooei‐Vayghan et al. 2020). Conversely, an increase in the feed rate led to a higher moisture content as it increased the water load entering the system. The water activity values of all samples (0.11–0.19) were well below the critical limit of 0.60 for microbial growth, indicating that the products will be microbiologically safe and possess a long shelf life. The moisture‐reducing effect of ultrasonication is significant, with the potential to lower drying costs.

The models for ash content, pH, and protein content were not significant (*p* > 0.05). Ash values ranged from 1.41% to 1.53%, and the protein content varied within a narrow band of 8.47 to 9.03 g/100 g. The applied treatments (temperature or ultrasonication) did not alter the inorganic matter content or cause macroscopic degradation of the macronutrients. The pH values remained neutral, ranging from 6.84 to 7.07. The invariability of the ash content, pH, and protein content is an expected and desirable outcome. This demonstrates that the applied ultrasonication and thermal treatments did not disrupt the fundamental mineral composition, acidity, or the basic nutritional baseline of the infant formula, thereby safely preserving its chemical stability. This finding supports the use of ultrasonication technology safely without compromising food safety or nutrient stability.

The findings at this stage of the study demonstrate that ultrasonication pre‐treatment significantly increased the process yield in infant formula production (an increase from approximately 12% up to 30%) and enabled the production of a drier product. While achieving these improvements, no adverse changes were observed in the fundamental chemical properties (ash, pH) of the product. These results substantiate that ultrasound pre‐treatment prior to spray drying could be a highly effective strategy to mitigate yield losses in the infant formula technology.

### 3.2 Powder Characterization

The reconstitution properties (solubility and wettability) of the infant formula powders obtained by spray drying are critical quality parameters regarding end‐user acceptability. Upon investigating the data obtained within the scope of the study, it was determined that ultrasonication time and inlet temperature had significant ameliorative effects on solubility and wettability (*p* < 0.05). In particular, the sample treated with a 12 mL/min feed rate, 180°C inlet temperature, and 90 s of ultrasonication reached the highest solubility value of 52.30%, while the wettability time decreased to 17 min. This phenomenon can be explained by the hydrodynamic shear forces exerted by ultrasonic cavitation on the macromolecules in the feed suspension. Acoustic cavitation disrupts protein aggregates and reduces the size of fat globules, thereby forming a more stable emulsion, which prevents the accumulation of free fat on the surface of the powder particles formed during drying. While the hydrophobic character of free fat on the powder surface hinders water penetration into the particle, the enhanced encapsulation of fat by proteins (protein migration to the surface due to the ultrasound effect) increased the powder's hydrophilicity, thereby shortening the wetting time. Furthermore, ultrasonication reduces feed viscosity, enabling the formation of smaller‐diameter droplets during atomization. This establishes a direct structure–function relationship: the significant reduction in final particle size parameters (D_4,3_ and D_3,2_) and the corresponding geometric increase in Specific Surface Area (SSA), as confirmed by laser diffraction (Section 3.5), dictate the reconstitution behavior. The maximized SSA provides a vastly larger solid–liquid interface, which accelerates water diffusion and capillary action, ultimately acting as the primary physical driver for the observed > 50% improvement in solubility and the significantly shortened wettability times. The increase in solubility with rising temperature can be attributed to particles formed at higher temperatures having a more porous crust structure, allowing water to penetrate the particle more rapidly through capillary action.

To properly contextualize these functional improvements, it is crucial to benchmark these values against existing literature. While conventional milk‐only infant formulas typically exhibit high solubility (> 90%), cereal‐based complementary foods, such as the complex matrix studied here containing wheat, rice, and oat flours, inherently possess much lower solubility indices (typically ranging from 30% to 45%) due to the presence of gelatinized starch networks and insoluble fiber fractions (Pasdar et al. 2024). In this context, achieving a solubility exceeding 50% and reducing wettability to 17 min represents a substantial processing advantage. Compared to standard, non‐ultrasonicated spray‐dried cereal powders that frequently suffer from severe lumping and prolonged wetting times, the ultrasound as pre‐treatment process demonstrates a clear functional superiority. By physically modifying the starch‐protein matrix, acoustic cavitation brings the reconstitution behavior of this complex complementary food closer to commercial instant‐grade standards without the need for additional chemical agglomerating agents.

When analyzing hygroscopicity values, a critical parameter for the storage stability of powder products, it was determined that hygroscopicity increased marginally as ultrasonication time increased. This increase can be explained by the reduction in particle size from ultrasonication, which increases the total surface area and expands the surface area in contact with ambient moisture. In systems containing lactose and low‐molecular‐weight sugars, such as infant formulas, the amorphous structure formed during spray drying possesses high hygroscopicity. It was found that ultrasonication facilitated water adsorption by increasing the proportion of amorphous regions on the particle surface. However, the hygroscopicity values of all samples remained within similar ranges, and no effect as pronounced as that of ultrasonication was observed on this parameter from either temperature or feed rate. This indicates that hygroscopicity is primarily governed by the chemical composition of the formulation and the glass transition dynamics (Tg) during drying, and that physical pre‐treatments can only alter this chemical affinity to a limited extent.

Considering the density (loose and tapped) and flowability indices (Carr Index and Hausner Ratio), which determine the flow properties and packaging behavior of powders, the obtained powders generally exhibited cohesive (sticky) and poor flowability (Table 3). The variation of CI values between 49.25% and 85.18% indicates that the powders possess highly compressible properties and that inter‐particle friction is prominent. However, it was determined that only ultrasonication time (C) had a significant effect on HR (*p* < 0.05). As the ultrasonication time increased, the HR and CI values partially decreased, indicating a limited but noticeable improvement in flowability. Mechanistically, this situation can be associated with the ultrasonication process modifying the agglomeration tendency. As stated by Song et al. (2022), ultrasonication can alter the particle size distribution, thereby affecting the packing arrangement of the particles. Nevertheless, in infant formula formulations with high fat and sugar contents, inter‐particle Van der Waals forces and liquid bridge formation are dominant; therefore, despite physical modifications, the powders remained in the poorly flowable category. Furthermore, it was observed that the loose bulk densities of the powders produced at high inlet temperatures (180°C) tended to decrease. This phenomenon can be explained by the principle that a high evaporation rate causes early crust formation on the droplet surface, and the vapor trapped within the particle expands, creating cavitation, which consequently increases intra‐particle porosity and decreases density. While the ultrasonication pre‐treatment significantly improved reconstitution properties, notably solubility and wettability, its effect on the flowability characteristics of the powder remained limited due to the nature of the formulation, although it possessed the potential to optimize packaging density.

**TABLE 3:** General factorial experimental design with three independent variables and their effects on loose density, tapped density, Carr index, and Hausner ratio of spray‐dried samples.

Run	A (°C)	B (mL/min)	C (s)	Loose density (g/cm^3^)	Tapped density (g/cm^3^)	Carr Index (CI, %)	Hausner Ratio (HR)
1	150	7	30	0.375 ± 0.005	0.586 ± 0.010	56.25 ± 4.88	1.5625 ± 0.048
2	180	7	30	0.361 ± 0.007	0.5565 ± 0.001	53.84 ± 3.31	1.5384 ± 0.033
3	150	12	30	0.363 ± 0.012	0.595 ± 0.009	63.93 ± 2.68	1.6393 ± 0.026
4	180	12	30	0.347 ± 0.017	0.579 ± 0.018	66.66 ± 2.77	1.6666 ± 0.027
5	150	7	60	0.361 ± 0.006	0.622 ± 0.010	72.41 ± 2.95	1.7241 ± 0.059
6	180	7	60	0.301 ± 0.006	0.558 ± 0.002	85.18 ± 3.43	1.8518 ± 0.034
7	150	12	60	0.317 ± 0.005	0.588 ± 0.012	85.18 ± 6.09	1.8518 ± 0.068
8	180	12	60	0.353 ± 0.003	0.620 ± 0.014	75.43 ± 6.16	1.7543 ± 0.061
9	150	7	90	0.333 ± 0.008	0.595 ± 0.004	78.57 ± 3.18	1.7857 ± 0.031
10	180	7	90	0.363 ± 0.009	0.541 ± 0.006	49.25 ± 2.22	1.4925 ± 0.022
11	150	12	90	0.364 ± 0.020	0.552 ± 0.024	51.51 ± 4.59	1.5151 ± 0.045
12	180	12	90	0.332 ± 0.018	0.519 ± 0.020	56.25 ± 2.44	1.5625 ± 0.024
Model *p*‐value				0.9807^ns^	0.3689^ns^	0.6614^ns^	0.0202*
Significant terms				nd	nd	nd	C
*R* ^2^				0.4159	0.9028	0.7861	0.5798
Adjusted *R* ^2^				−2.2128	0.4653	−0.1763	0.4864
Predicted *R* ^2^				−20.0290	−2.4999	−6.6992	0.2530
Adeq Precision				1.53	4.01	2.59	4.45
C.V. (%)				11.40	3.99	21.29	5.61
Standard deviation				0.0397	0.0230	14.11	0.0933

*Note:* Values are expressed as mean ± standard deviation. All analyses were performed at least in triplicate. The experimental design consisted of a general factorial design with 12 experimental runs. Independent variables were spray dryer inlet temperature (150°C and 180°C), feed rate (7 and 12 mL/min), and ultrasonication time (30–90 s). Goodness‐of‐fit measures for each response are listed at the bottom of the table.

Abbreviations: A, Spray dryer inlet temperature (°C); B, feed rate (mL/min); C, ultrasonication time (s); nd, not determined.

* Indicates a significant model at *p* < 0.05.

ns: non‐significant (*p* > = 0.05).

### 3.3 Color

The visual properties of infant formula powders obtained via spray drying provide critical data regarding the sensory acceptability of the product and the extent of thermal deformation occurring during the process. Upon examining the colorimetric data obtained within the scope of the study (Table 4), it was observed that the powders characteristically possessed high lightness (L* > 92.0) and low chroma values, thereby maintaining the desired white/cream color. It was revealed that ultrasonication time (C) was the primary determining factor for the L* value (*p* < 0.05), and that lightness was preserved or marginally increased with higher ultrasonic energy input. Mechanistically, this phenomenon can be explained by the hydrodynamic shear forces caused by ultrasonic cavitation, which reduce the fat globules and protein aggregates in the feed suspension to sub‐micron sizes. This reduction in particle size increases the specific surface area of the dried powder, thereby elevating the light scattering coefficient and increasing the opacity and lightness perception of the material in accordance with the Lambert–Beer law. The smoother and smaller particle morphology obtained through the ultrasonic homogenization effect promotes the reflection of light from the surface rather than its absorption, thus increasing the L* value (Tong et al. 2022).

**TABLE 4:** General factorial experimental design with three independent variables and their effects on color parameters and browning index of spray‐dried samples.

Run	A (°C)	B (mL/min)	C (s)	L*	a*	b*	Chroma	Hue angle (°)	Browning Index (BI)
1	150	7	30	92.99 ± 1.57	−1.20 ± 0.05	5.56 ± 0.38	5.69 ± 0.24	102.23 ± 0.95	5.01 ± 0.36
2	180	7	30	92.98 ± 1.10	−1.20 ± 0.11	5.49 ± 0.65	5.59 ± 0.19	102.40 ± 1.02	4.93 ± 0.74
3	150	12	30	92.82 ± 1.02	−1.26 ± 0.02	5.58 ± 0.47	5.72 ± 0.09	102.71 ± 1.10	4.99 ± 0.47
4	180	12	30	93.04 ± 0.89	−1.24 ± 0.09	5.58 ± 0.91	5.71 ± 0.31	101.86 ± 0.63	4.99 ± 1.02
5	150	7	60	93.27 ± 1.10	−1.19 ± 0.17	5.32 ± 0.16	5.45 ± 0.47	102.61 ± 0.54	4.73 ± 0.24
6	180	7	60	93.28 ± 1.21	−1.17 ± 0.04	5.86 ± 0.99	5.98 ± 0.07	101.36 ± 0.39	5.34 ± 1.04
7	150	12	60	93.39 ± 0.87	−1.15 ± 0.07	5.19 ± 0.14	5.40 ± 0.18	102.27 ± 0.01	4.62 ± 0.16
8	180	12	60	93.19 ± 0.84	−1.19 ± 0.04	5.68 ± 0.76	5.80 ± 0.27	101.85 ± 0.37	6.98 ± 0.80
9	150	7	90	92.99 ± 0.91	−1.17 ± 0.07	5.48 ± 0.21	5.61 ± 0.15	102.02 ± 0.91	4.94 ± 0.23
10	180	7	90	92.96 ± 0.56	−1.20 ± 0.03	5.47 ± 0.38	5.60 ± 0.12	102.35 ± 0.53	4.91 ± 0.41
11	150	12	90	92.83 ± 0.74	−1.17 ± 0.11	5.62 ± 0.47	5.75 ± 0.42	101.73 ± 0.48	5.10 ± 0.56
12	180	12	90	93.03 ± 1.09	−1.18 ± 0.02	5.47 ± 0.27	5.60 ± 0.23	102.18 ± 0.17	4.92 ± 0.25
Model *p*‐value				0.0099**	0.5652^ns^	0.0197*	0.1365 ^ns^	0.7515^ns^	0.4713^ns^
Significant terms				C	None	A, AC	AC	None	None
*R* ^2^				0.8799	0.8311	0.9786	0.9679	0.7339	0.8679
Adjusted *R* ^2^				0.7799	0.0708	0.9217	0.8235	−0.4637	0.2737
Predicted *R* ^2^				0.5198	−5.0820	0.6583	−0.1550	−8.5806	−3.7540
Adeq Precision				7.12	3.93	16.31	9.77	2.14	4.72
C.V. (%)				0.09	66.73	0.86	1.15	0.46	10.17
Standard deviation				0.0844	0.6640	0.0474	0.0650	0.4666	0.5210

*Note:* Values are expressed as mean ± standard deviation. All analyses were performed at least in triplicate. The experimental design consisted of a general factorial design with 12 experimental runs. Independent variables were spray dryer inlet temperature (150°C–180°C), feed rate (7–12 mL/min), and ultrasonication time (30–90 s). Goodness‐of‐fit measures for each response (L*, a*, b*, Chroma, Hue angle, and Browning Index) are listed at the bottom of the table.

Abbreviations: A, Spray dryer inlet temperature (°C); B, feed rate (mL/min); C, ultrasonication time (s).

* Indicates a significant model at *p* < 0.05.

** Indicates a significant model at *p* < 0.01; ns, non‐significant (*p* ≥ 0.05).

When examining the b* and a* values, the thermal and chemical dynamics of the process present a more complex outcome. The a* values remained in the negative region (tending toward greenness) and did not show significant changes (*p* > 0.05), which is associated with the absence of red‐brown pigments typically formed because of caramelization or advanced Maillard reactions. However, a significant effect of Temperature (A) and the A × C interaction on the b* value was determined (*p* < 0.05). The variation observed in the b* value with increasing temperature is related to the formation of Schiff bases between lactose and lysine residues in the initial stage of the Maillard reaction, followed by their transformation into Amadori products. Amadori products are colorless or light yellow and are primarily responsible for the positive shift on the b* axis. The regulatory role of ultrasonication in this process (A × C interaction) can be associated with the local temperature increases and free radicals (OH·) generated by the collapse of cavitation bubbles, which modify glycation kinetics by lowering the reaction activation energy. The unfolding of the protein structure by ultrasonication, thereby exposing reactive groups, may have limitedly promoted yellow pigment formation when combined with high temperatures.

The non‐significance (*p* > 0.05) of the model determined for the Browning Index (BI), a key indicator of product quality, indicates that within the studied parameter range (150°C–180°C), browning reactions remained in the diffusion‐controlled region and did not progress to the advanced stages of non‐enzymatic browning. This situation can be explained by the thermodynamic nature of the spray drying process: the rapid evaporation of water from the droplet surface ensures that the particle temperature is stabilized at the wet‐bulb temperature. When the particle is exposed to the high temperature of the drying medium, its moisture content drops below the critical glass transition concentration, and the system enters the glassy phase (Tg), thereby restricting molecular mobility. The reduction in molecular mobility prevents the diffusion of reactants toward each other, thus halting the formation of brown polymers such as melanoidins. Consequently, the ultrasonication pre‐treatment improved the lightness of the product by reducing particle size and increasing light reflection capacity; when combined with the short residence time of the process, it minimized thermal damage and provided a high‐quality visual profile, resulting in a product that avoided oxidative and thermal degradation.

### 3.4 Amadori Compounds, Melanoidins, and HMF

In heat‐treated infant formula formulations, the Maillard reaction, triggered by the interaction between reducing sugars and the amino groups of proteins, is among the primary chemical mechanisms determining the nutrient composition and toxicological safety of the product. Within the scope of this study, the initial (Amadori compounds), intermediate, and advanced (melanoidins and HMF) stages of the Maillard reactions were determined. The effects of process parameters on these reaction steps were evaluated using the data presented in Table 5. The results indicate that while the model established for the formation of colorless Amadori compounds, representing the early stage of the Maillard reaction, was non‐significant (*p* > 0.05), the individual process variables (A, B, C) were identified as effective factors in the model (*p* < 0.05). The variation of absorbance values between 1.09 and 2.59 confirms the occurrence of Schiff base formation followed by Amadori rearrangement in the lactose‐ and protein‐rich formulation. Notably, Amadori compounds reached their maximum level (2.595) in samples where the highest inlet temperature (180°C), highest feed rate (12 mL/min), and longest ultrasonication time (90 s) were applied. This phenomenon can be explained by the increased heat load, which enhances the kinetic energy of the reactants and accelerates carbonyl‐amino condensation. Furthermore, the unfolding of the tertiary structure of proteins due to the ultrasonic cavitation effect may have rendered reactive amino groups more accessible, thereby increasing the probability of interaction with lactose.

**TABLE 5:** General factorial experimental design with three independent variables and their effects on Amadori compounds, melanoidins, and HMF formation of spray‐dried samples.

Run	A (°C)	B (mL/min)	C (s)	Absorbance at 304 nm (Amadori)	Absorbance at 420 nm (Melanoidins)	HMF (mg/kg)
1	150	7	30	1.1833 ± 0.08	0.7120 ± 0.09	33.4 ± 1.12
2	180	7	30	1.4782 ± 0.11	0.4955 ± 0.10	53.0 ± 2.11
3	150	12	30	1.7835 ± 0.14	1.0101 ± 0.14	39.5 ± 1.15
4	180	12	30	2.0431 ± 0.18	1.1468 ± 0.09	46.6 ± 1.10
5	150	7	60	1.3836 ± 0.09	0.8658 ± 0.07	41.5 ± 2.05
6	180	7	60	1.7458 ± 0.16	1.0018 ± 0.08	54.4 ± 3.10
7	150	12	60	1.0913 ± 0.05	0.4673 ± 0.07	47.9 ± 2.56
8	180	12	60	1.8923 ± 0.17	1.0371 ± 0.11	43.1 ± 1.55
9	150	7	90	1.5880 ± 0.07	0.8305 ± 0.07	31.0 ± 2.19
10	180	7	90	2.0730 ± 0.16	1.1192 ± 0.14	41.1 ± 2.05
11	150	12	90	2.0137 ± 0.14	1.0632 ± 0.06	54.3 ± 3.23
12	180	12	90	2.5950 ± 0.21	1.1276 ± 0.09	54.5 ± 3.50
Model *p*‐value				0.0662^ns^	0.3895^ns^	0.4028 ^ns^
Significant terms				A, B, C	None	None
*R* ^2^				0.9849	0.8962	0.8918
Adjusted *R* ^2^				0.9169	0.4288	0.4048
Predicted *R* ^2^				0.4563	−2.7385	−2.8962
Adeq Precision				13.01	4.28	4.06
C.V. (%)				7.04	19.80	13.95
Standard deviation				0.1224	0.1795	6.28

*Note:* Values are expressed as mean ± standard deviation. All analyses were performed at least in triplicate. The experimental design consisted of a general factorial design with 12 experimental runs. Independent variables were spray dryer inlet temperature (150°C–180°C), feed rate (7–12 mL/min), and ultrasonication time (30–90 s). Goodness‐of‐fit measures for each response are listed at the bottom of the table.

Abbreviations: A, spray dryer inlet temperature (°C); B, feed rate (mL/min); C, ultrasonication time (s); ns, non‐significant (*p* ≥ 0.05).

When examining the advanced stage indicators of the reaction, namely melanoidins and HMF, it was determined that the models generated for both parameters were non‐significant (*p* > 0.05), and the independent variables did not create a decisive variation in these advanced glycation products. The fact that HMF absorbance values remained within a narrow band is associated with the short residence time characteristic of the spray drying process. In spray drying, the rapid evaporation of water from the droplet surface ensures that the particle temperature remains at the wet‐bulb temperature. The particle is exposed to the high temperature of the drying air only when the moisture content drops below a critical level. At this stage, the system transitions into the glassy phase, restricting molecular mobility, and the reaction reaches a standstill as it becomes diffusion‐controlled. Consequently, even if the ultrasonication time or inlet temperature is increased, it is understood that the reaction does not find sufficient time to progress to the advanced stages required to form HMF or melanoidins.

Regarding the effect of ultrasonication time, although the local temperature increases caused by the collapse of cavitation bubbles theoretically pose a risk of triggering the Maillard reaction, the non‐significance of the HMF and melanoidin models in this study suggests that this effect was tolerated within the dominant thermal dynamics of spray drying. Since direct measurements of Maillard reaction kinetics were not performed in this study, the exact mechanistic role of ultrasound cannot be definitively concluded. However, we hypothesize that rather than exerting a direct chemical inhibition, ultrasonication indirectly limited the formation of advanced glycation products through physical means. By reducing feed viscosity and generating finer atomized droplets, the pre‐treatment accelerated the evaporation rate. This physical effect is hypothesized to have shortened the critical duration during which the particles retained high moisture under thermal stress, effectively shifting the sucrose‐protein matrix into a low‐mobility glassy state before the reaction could progress to the advanced HMF stage. This is supported by the fact that HMF values remained at low levels in some samples with high ultrasonication times. It was determined that the infant formula formulation exhibited chemical stability regarding the Maillard reaction under the examined process conditions. The reaction products remained largely limited to early‐stage compounds, and HMF accumulation, as a source of toxicological concern, was kept under control regardless of the process parameters. This finding supports the definition of ultrasound pre‐treatment prior to drying as a reliable technology for the food safety and sensory quality of infant formulas.

### 3.5 Particle Size Distribution

In spray drying technology, the reconstitution ability, flowability, and packaging properties of the final product are largely determined by the particle size distribution and surface morphology. The laser diffraction data obtained (Table 2) demonstrate that ultrasonication pre‐treatment exerted a distinct and statistically significant effect (*p* < 0.05) on reducing particle diameters (D_3,2_ and D_4,3_) and consequently increasing the Specific Surface Area (SSA). While the Sauter Mean Diameter (D_3,2_) is a parameter sensitive to the surface area of the particle and reflects atomization efficiency, the Volume Weighted Mean Diameter (D_4,3_) points to the presence of coarse particles or agglomerates in the system. The fundamental mechanism behind the decrease observed in both diameter values with increasing ultrasonication time is the acoustic cavitation phenomenon generated within the liquid feed, which is directly validated by the empirical trends reported in Table 2. For instance, at a constant inlet temperature of 180°C and a feed rate of 7 mL/min, extending the ultrasonication duration from 30 s (Run 2) to 60 s (Run 6) resulted in a direct quantitative reduction of the volume‐weighted mean diameter (D_4,3_) from 7.34 ± 0.71 m to 6.24 ± 0.11 m, while the specific surface area (SSA) expanded from 817.5 ± 5.20 to 961.7 ± 12.0 m^2^/kg. High‐intensity sound waves cause the formation and subsequent violent collapse of microscopic vapor bubbles in the liquid medium (Chemat and Khan 2011). The high hydrodynamic shear forces and turbulence released during this collapse are hypothesized to physically disrupt the macromolecular networks and fat globules in the complex infant formula matrix. Although direct rheological measurements of the feed suspension were not conducted in this study, the significant decrease in the final powder particle size values serves as a strong macroscopic proxy, inferring a reduction in feed viscosity. This inferred reduction facilitates the breakup of the liquid at the atomizer nozzle, increasing the Weber number and resulting in much finer and more homogeneous droplets being sprayed into the drying chamber (Song et al. 2022).

This atomization enhancement via cavitation is further confirmed under high feed rate conditions (12 mL/min) at 180°C, where prolonging the treatment from 30 s (Run 4) to 90 s (Run 12) successfully decreased the Sauter mean diameter (D_3,2_) from 20.3 ± 2.04 to 19.2 ± 0.65 m, while simultaneously driving a reduction in D_4,3_ from 7.93 ± 0.35 to 7.10 ± 0.59 m. In terms of drying kinetics, smaller initial droplets dry more rapidly due to enhanced heat and mass transfer, transforming into smaller solid particles, which explains the consistent decline in diameter values. On the other hand, the reduction in the D_4,3_ value reflects the de‐agglomeration efficiency of ultrasonication. Due to their high protein and sucrose content, infant formulas tend to form large agglomerates when emulsion stability is compromised or through coalescence during drying. Ultrasonic shock waves disperse protein aggregates in the feed solution and reduce fat globules to sub‐micron sizes, creating a more stable emulsion structure (Song et al. 2022). This prevented random particle coalescence during the spray drying process, directly explaining why the physical data exhibit a consistent narrowing of the particle size distribution and a geometric increase in SSA values as the mechanical energy input increases. The SSA maximized by ultrasonication offers a critical advantage in terms of the functional properties of the powder. The increased surface area accelerates the diffusion of water molecules to the particle‐water interface, thereby improving the powder's wettability. This finding is consistent with the higher solubility rates in ultrasonicated samples discussed in the previous sections regarding reconstitution properties (Schutyser et al. 2019). Although previous studies have suggested that very small particles might lead to flowability issues due to increased cohesive forces (Boonyai et al. 2004), the particle size range obtained via ultrasonication in our study indicates an optimal balance that enhances solubility without compromising flowability to an intolerable degree. Therefore, the ultrasonication pre‐treatment modified the feed rheology and emulsion structure through the mechanism of hydrodynamic cavitation, minimizing D_3,2_ and D_4,3_ values by increasing atomization efficiency. This structural modification yielded a morphologically homogeneous powder with increased SSA, resulting in rapid dissolution and high functional quality.

### 3.6 Micro‐Structure

**FIGURE 1:**
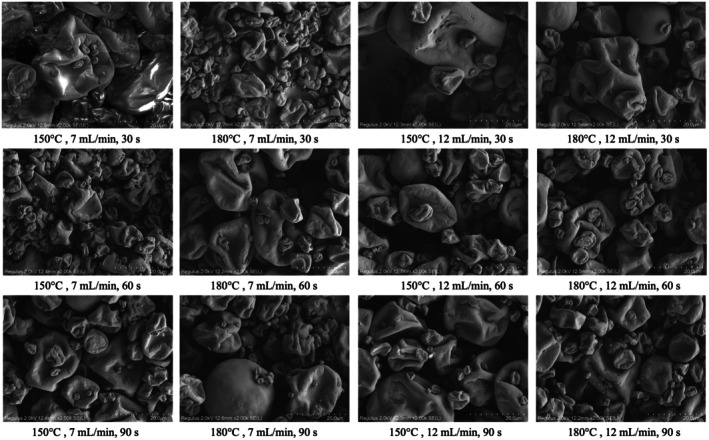
Scanning electron microscopy (SEM) micrographs of spray‐dried infant formula powders produced under varying process conditions. Labels indicate the specific inlet air temperature (°C), feed rate (mL/min), and ultrasonication pre‐treatment time (s) applied for each experimental run.

The microstructural properties, surface morphology, and particle size characteristics of the spray‐dried infant formula powders were evaluated using Scanning Electron Microscopy (SEM) (Figure 1). The micrographs revealed that the powders generally exhibited a predominantly spherical morphology with varying degrees of surface indentations (concavities) and agglomeration, which are typical structural characteristics of spray‐dried carbohydrate‐ and protein‐rich dairy matrices (Murrieta‐Pazos et al. 2012). The morphological variations observed among the samples provide profound insights into the interactions among the independent variables (ultrasonication time, inlet temperature, and feed rate) and the powders' physical stability.

The application and prolongation of ultrasonication (US) pre‐treatment (from 30 to 90 s) exerted a distinct modifying effect on particle morphology and size. SEM observations corroborated the laser diffraction data, showing that samples subjected to longer ultrasonication exhibited noticeably smaller and more uniformly distributed individual particles with a reduced tendency for large‐scale agglomeration. The acoustic cavitation induced by the ultrasonic probe is inferred to have effectively disrupted protein aggregates and homogenized the oil‐in‐water emulsion within the feed suspension prior to atomization (Chemat and Khan 2011). Consequently, the uniform dispersion of sub‐micron fat globules is proposed to facilitate a more continuous and stable protein‐carbohydrate matrix upon drying. From a stability perspective, this smoother and less agglomerated surface morphology indirectly suggests an enhanced encapsulation efficiency of lipids, which would theoretically minimize the presence of surface free fat, which is critical for delaying lipid oxidation and improving the oxidative stability of the infant formula during shelf life (Fäldt and Bergenståhl 1995).

Inlet air temperature was identified as another major driving force dictating the surface topography of the particles. Powders produced at a lower inlet temperature (150°C) displayed more pronounced surface shrinkage and deeper dents. This morphological behavior is attributed to the slower evaporation rate, which allows the droplet surface to remain flexible for a longer period, leading to the particle deflating and shriveling as the internal pressure decreases during cooling (Nijdam and Langrish 2006). Conversely, at a higher inlet temperature (180°C), rapid water evaporation leads to the early formation of a rigid crust on the droplet surface (case hardening). As the trapped internal moisture vaporizes and expands, it exerts outward pressure against this rigid crust, leading to a balloon effect (Soottitantawat et al. 2005). As a result, particles dried at 180°C appeared more spherical, smoother, and slightly larger in apparent volume, albeit internally hollow or highly porous. This rigid spherical structure is highly advantageous for powder stability, as it prevents particle collapse and inter‐particle bridging.

The feed rate significantly influenced the agglomeration behavior and microstructural stability. At the highest feed rate (12 mL/min) combined with the lower inlet temperature (150°C), the SEM micrographs clearly displayed evident particle clustering, liquid bridges, and fused surfaces (caking). High feed rates increase the droplet size and the total water load entering the drying chamber, resulting in particles with higher residual moisture. As discussed in the thermal behavior analysis (Section 3.7), this residual moisture acts as a strong plasticizer, depressing the glass transition temperature (Tg) below the ambient temperature. When the particle surface remains in a rubbery state rather than a glassy state, particles colliding in the drying chamber or cyclone fuse together, leading to irreversible agglomeration and structural collapse (Boonyai et al. 2004). The microstructural evaluation demonstrates a strong interaction among the process variables. The optimization of ultrasonication time provided finer, more homogeneous droplets with improved fat encapsulation, while higher inlet temperatures ensured rapid crust formation, establishing a rigid, glassy barrier. Together, these conditions mitigated stickiness and caking, thereby establishing a highly stable microstructural geometry that protects the sensitive nutritional components of the infant formula against physical and chemical degradation.

While these static microstructural profiles offer vital visual insights into the final powder geometry, they do not provide direct empirical evidence of active cavitation or dynamic phase changes within the liquid feed stream. Because direct measurements of pre‐drying feed rheology, emulsion stability, and atomized droplet size distributions were not performed in this study, characterizing these morphological transitions as a direct consequence of ultrasound‐induced cavitation remains a mechanistic hypothesis rather than a directly monitored event.

Nonetheless, this structural interpretation is supported semi‐quantitatively by a critical convergence across our other physical datasets. The significant reduction in final volumetric and surface mean diameters, confirmed by independent laser diffraction measurements (Table 2), serves as a strong macroscopic proxy, strongly indicating that the mechanical energy input from the acoustic field successfully altered the fluid's resistance to atomization at the nozzle. This atomization enhancement is further reflected in the accelerated drying kinetics inferred by the final powder characteristics: the significantly lower residual moisture content (Table 1) and the corresponding upwards shift in glass transition temperature in ultrasonicated runs suggest a faster transition into the rigid amorphously stable phase before excessive particle‐wall collisions or random particle coalescence could take place. Therefore, although dynamic fluid phenomena cannot be directly visualized, the coupled particle, thermal, and morphological trends align structurally with the proposed hypothesis of ultrasound‐modulated feed configuration.

### 3.7 Thermal Behavior

**FIGURE 2:**
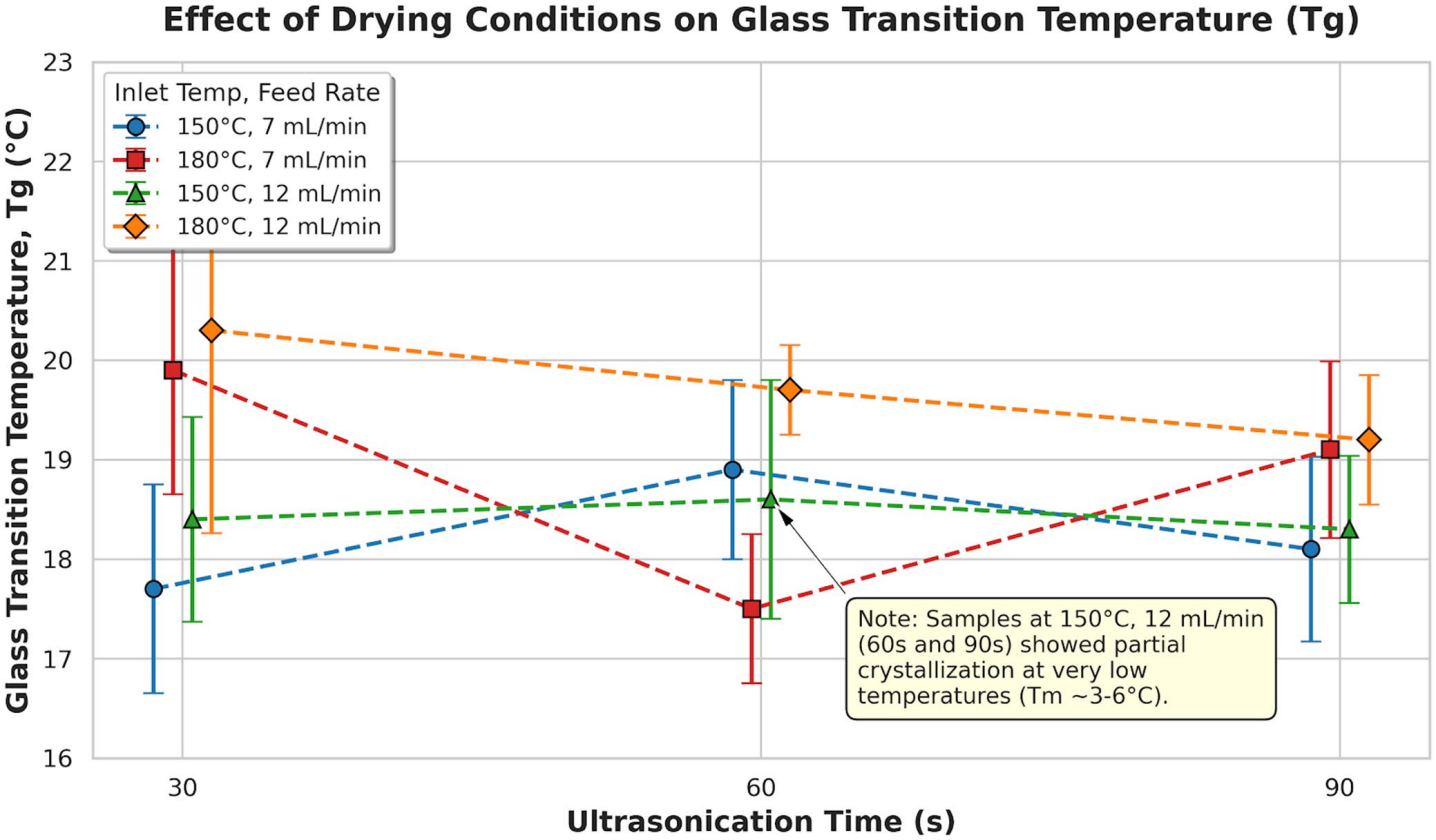
Effect of spray drying parameters (inlet air temperature, feed rate, and ultrasonication time) on the glass transition temperature (Tg) of the infant formula powders. Error bars represent the standard deviation (±SD) of the mean. Samples processed at 150°C and 12 mL/min (with 60 and 90 s ultrasonication) exhibited partial crystallization/melting behavior (Tm ~3°C–6°C) at very low temperatures rather than a distinct amorphous relaxation.

In systems containing lactose and low‐molecular‐weight sugar derivatives, such as infant formulas, the glass transition temperature (T_g_) is one of the most critical thermal parameters determining the physical stability, flowability, and shelf life of the product. During the spray drying process, if the temperature in the drying medium exceeds the Tg of the particles, the product transitions from a glassy state to a rubbery state. This transition increases inter‐particle cohesion, leading to the stickiness problem (Boonyai et al. 2004; Palzer 2005). Upon examining the obtained DSC thermograms, it was observed that the powders produced under most drying conditions (inlet temperatures of 150°C–180°C, feed rates of 7–12 mL/min, and ultrasonication intervals of 30–90 s) generally exhibited a broad endothermic enthalpy relaxation peak between 29°C and 38°C. This thermal behavior is a typical indicator of the amorphous nature of spray‐dried high‐sugar food powders and confirms that the product was largely dried in a glassy state (Bhandari et al. 1997). According to the data in Figure 2, the glass transition temperatures of the amorphous powder samples ranged from 17.5°C to 20.3°C. These thermal properties establish a critical structure–function relationship that directly explains the macroscopic processing behavior of the formula. The relatively low glass transition temperatures (T_g_) observed in this study quantitatively governed the stickiness dynamics and, consequently, the restricted process yield reported in Section 3.1. Since the inlet temperatures (150°C–180°C) remained substantially above the T_g_ of the samples, the particles likely existed in a highly cohesive, low‐viscosity rubbery state. This thermodynamic state (T>T_g_) acts as the mechanistic cause for the inter‐particle liquid bridge formation and strong adherence to the cyclone and chamber walls, physically validating why the baseline powder collection yields remained challenging without the application of ultrasound. This phenomenon is a well‐known challenge in spray drying high‐sugar and protein‐rich formulations without the use of excessive carrier agents.

When evaluating the effects of process parameters on T_g_, it is evident that the residual moisture within the matrix exerts a very strong plasticizing effect, which is directly linked to the physical data reported in Table 1. Although the water activity values of all samples remained exceptionally low (0.1113–0.1934), even a minimal amount of residual moisture content (1.46–2.73 g/100 g) acts as a powerful plasticizer due to the high hygroscopicity of the amorphous lactose and sucrose fractions within the composite matrix. For instance, in samples where a 7 mL/min feed rate and 30‐s ultrasonication were applied, increasing the inlet air temperature from 150°C to 180°C enhanced evaporation kinetics and reduced the moisture content from 1.95% to 1.48%, respectively. This weakened the plasticizing effect of water and raised the T_g_ of the matrix from 17.7°C to 19.9°C. From a practical and industrial standpoint, these low T_g_ values (17.5°C–20.3°C) carry vital implications for the storage stability and commercial shelf life of the final product. Since standard ambient storage and distribution conditions (≈25°C) remain systematically above the developed T_g_ window (T>T_g_), the amorphous powder is highly prone to experiencing a rapid glassy‐to‐rubbery state transition over time. In this rubbery state, the accelerated molecular mobility and decreased viscosity at the particle surface promote spontaneous moisture adsorption, inter‐particle liquid bridge formation, and eventual caking or structural collapse. Consequently, to safeguard the physical integrity and reconstitution quality of this complex cereal–milk formulation, it is critically imperative to implement specific post‐processing controls. These industrial guidelines include strictly storing the packaged powders under suitable conditions below their thermal threshold and deploying advanced multi‐layer hermetic packaging materials with superior water‐vapor transmission rate (WVTR) barriers to block environmental humidity ingress and prevent caking‐induced degradation.

Similarly, increasing the ultrasonication time provided a structural improvement in Tg stability. The acoustic cavitation phenomenon generated by ultrasonication in the liquid medium reduces the feed viscosity and decreases the droplet size formed during atomization (Chemat and Khan 2011; Song et al. 2022). The smaller droplet diameter increased the heat and mass transfer surface area, facilitating the reduction of moisture content below the critical level before the particles reached the dryer walls, thereby supporting their fixation in the amorphous phase.

Interestingly, samples processed under the combination of low inlet temperature (150°C) and high feed rate (12 mL/min) with prolonged ultrasonication did not exhibit a distinct amorphous relaxation (T_g_). Instead, these samples displayed endothermic transitions indicative of melting (T_m_ ~3°C–6°C), suggesting the occurrence of partial crystallization driven by the strong plasticizing effect of high residual moisture. Although absolute quantitative crystallinity was not determined via X‐ray diffraction, this transition from an amorphous state to a semi‐crystalline structural matrix is semi‐quantitatively corroborated by the molecular data. Specifically, the significant shifts observed in the FTIR 1022/995 cm^−1^ absorbance ratios (please see Section 3.8) confirm an increase in the short‐range molecular order of the starch and sucrose fractions. Thus, the coupled thermal and molecular data robustly indicate the formation of localized semi‐crystalline domains, even without absolute XRD quantification.

### 3.8 Molecular Characterization by FTIR

**FIGURE 3:**
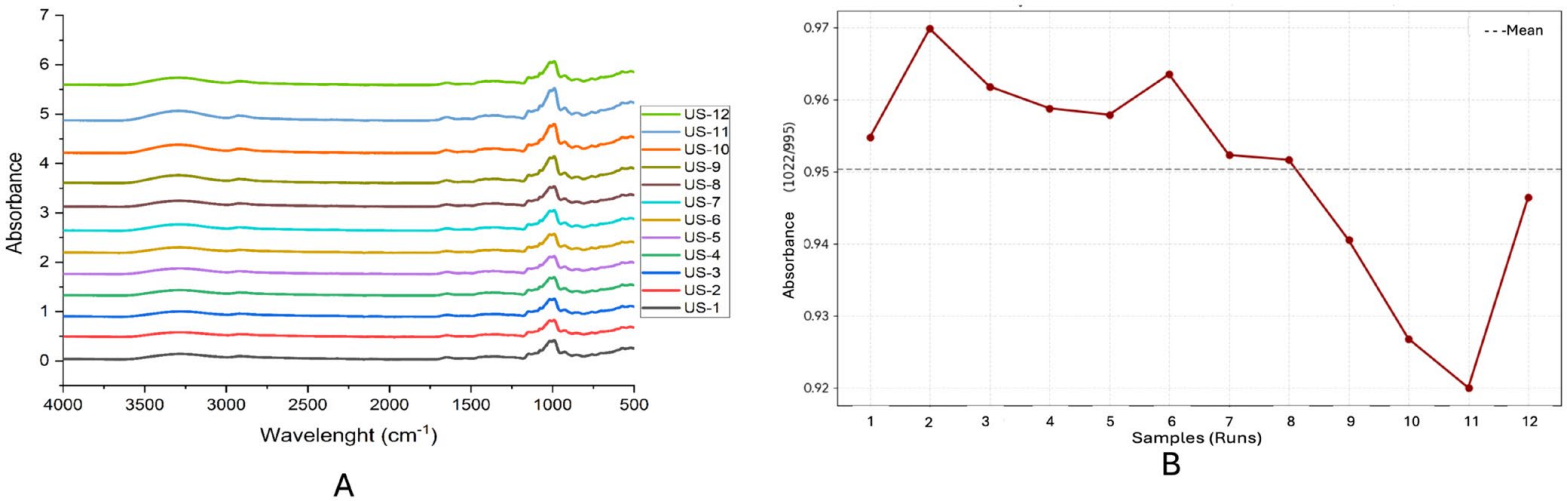
FTIR spectra of the spray‐dried infant formula powders under different ultrasonication and drying conditions. (A) The complete mid‐infrared region showing major functional groups (O‐H, N‐H, and C‐H stretching) and the Amide I band. (B) Magnified view of the fingerprint region (900–1150 cm^−1^) highlighting the molecular rearrangement and changes in the amorphous‐to‐crystalline ratio (1022/995 cm^−1^) of the starch matrix.

The structural changes at the molecular level, protein secondary structures, and the effects of process parameters on inter‐component interactions in the infant formula formulations were investigated using Fourier Transform Infrared (FTIR) spectroscopy (Figure 3). The specific absorption bands observed in the acquired spectra reflect the thermomechanical effects of the applied spray drying and ultrasonication pre‐treatment on the product matrix (Sun et al. 2018). The broad absorption band in the 3000–3600 cm^−1^ region of the spectra represents the O–H stretching of free and bound water in the system, as well as the N–H stretching in protein molecules (Tong et al. 2022). The relative increase in absorbance intensity in this region for the trials where the longest ultrasonication time (90 s) was applied suggests that acoustic cavitation may have induced tentative alterations in the local conformation of the proteins, which could be attributed to a partial unfolding behavior that potentially exposes hydrophilic groups to the surface; this, in turn, is hypothesized to have the water‐holding capacity and the intra‐ and intermolecular hydrogen bonding interactions (Chemat and Khan 2011). The weak peaks observed in the 2800–3000 cm^−1^ range are primarily attributed to the aliphatic C–H stretching of lipids and the carbohydrate skeleton (Sun et al. 2018). The absence of enhanced differences in this region, despite changes in drying parameters, suggests that the applied thermal treatment and ultrasonication energy did not disrupt the fundamental lipid stability of the product and did not lead to major oxidative or chemical degradation.

Upon examining the Amide I region (1600–1700 cm^−1^), which is the most critical area for protein characterization and provides information about the protein secondary structure (α‐helix, β‐sheet), a stable shoulder/peak was observed around 1640–1650 cm^−1^ (Zhang et al. 2022). The low variation in intensity in this region reflects the general stability of the macro‐colloidal stream. Upon examining the Amide I region (1600–1700 cm^−1^), which is highly sensitive to the local conformation of peptide backbones (Zhang et al. 2022), a visually consistent shoulder and peak profile was observed around 1640–1650 cm^−1^. Because rigorous mathematical deconvolution and quantitative curve‐fitting of individual secondary structure percentages were not within the scope of this study, absolute preservation of the protein secondary structures cannot be definitively asserted. However, the qualitative overlap and the absence of pronounced peak broadening or major band shifts in this primary amide zone strongly imply that the combined ultrasound pre‐treatment intervals and spray drying temperatures (150°C–180°C inlet) did not induce extensive or catastrophic macro‐scale thermal denaturation of the core protein architecture (Tong et al. 2022). The 900–1150 cm^−1^ range (fingerprint region), where the most dominant absorptions were observed, reflects the C–O and C–C stretching of the formulation, which is highly rich in starch, lactose, and sucrose (Sun et al. 2018). In particular, the prominent peaks in the 1000–1020 cm^−1^ band confirm the dense carbohydrate content of the matrix. The intensification of this peak in samples subjected to a long ultrasonication time (90 s) suggests that high cavitation forces modulated the short‐range molecular reordering and local conformation (Chemat and Khan 2011).

To investigate the molecular order of starch (short‐range order) more deeply, the ratio of the peak at 1022 cm^−1^, representing the amorphous (disordered) phase, to the peak at 995 cm^−1^, representing the double‐helical crystalline (ordered) structure, was calculated (1022/995) (Sevenou et al. 2002). An increase in this ratio indicates the predominance of the amorphous structure, whereas a decrease signifies an increase in crystalline structure and short‐range order due to molecular rearrangement (Warren et al. 2016). The absorbance ratios obtained from the FTIR spectra suggest that the short‐range molecular order of the carbohydrate fractions (starch and sucrose) was dynamically modulated, rather than completely transformed, depending on the intensity of the pre‐treatment. The highest ratio (0.9698) obtained in the sample dried at the shortest ultrasonication time (30 s), low feed rate (7 mL/min), and highest inlet temperature (180°C) indicates that rapid evaporation kinetics and short cavitation time fixed the starch matrix in a highly amorphous character during the initial stage (Palzer 2005). In contrast, this ratio decreased to its lowest level (0.9200) in the sample processed with the longest ultrasonication time (90 s), high feed rate (12 mL/min), and low inlet temperature (150°C). High system moisture (originating from low temperature and high feed rate) combined with prolonged mechanical energy input created a plasticizing effect in the matrix, thereby increasing the short‐range molecular order (Warren et al. 2016). This structural finding is in significant agreement with the partial crystallization/melting (Tm) behavior observed at low temperatures under the exact same process conditions in the differential scanning calorimetry (DSC) analyses.

## 4 Conclusion

In this study, the effects of an ultrasound pre‐treatment applied prior to conventional spray drying on the process efficiency and final product quality of a model cereal–milk infant formula formulation were investigated using a holistic approach. The findings revealed that utilizing ultrasonication as a non‐thermal pre‐treatment on the liquid feed offers a significant strategy to solve fundamental technological challenges, such as low yield, particle stickiness, and poor solubility issues. The most striking result of the study is the distinct positive effect of ultrasonication on drying yield. Increasing the ultrasonication duration reduced the viscosity of the feed suspension and enhanced atomization efficiency, thanks to the hydrodynamic shear forces generated by acoustic cavitation. This facilitated the formation of smaller and more homogeneous droplets, minimizing product accumulation on the drying chamber walls and increasing the yield from approximately 12%–30%.

Regarding the reconstitution properties of the powder product, the ultrasonication process significantly reduced particle size (D_3,2_ and D_4,3_), thereby increasing the specific surface area (SSA). The combination of an expanded surface area and more effective encapsulation of fat globules by ultrasonication shortened the wetting time and raised solubility values above 50%. This improvement represents a critical quality enhancement in meeting end‐user expectations for ease of preparation. When examining Maillard reaction products, as the most sensitive parameters in terms of food safety, no significant increase was observed in the formation of advanced glycation products such as HMF and melanoidins, despite the energy input generated by ultrasonication. On the contrary, the drying kinetics accelerated by ultrasonication may have limited chemical degradation by shortening the duration during which particles are exposed to high moisture and high temperature simultaneously. The produced powders maintained HMF levels considered safe within legal limits. Furthermore, the ultrasonication treatment improved the optical properties of the powders, ensuring the preservation of the desired lightness (L*) values. In conclusion, samples prepared with increased ultrasound pre‐treatment duration and higher spray dryer inlet temperatures exhibited high process efficiency, improved solubility, and preserved chemical stability. This study concludes that the strategic integration of an ultrasound pre‐treatment stage into industrial infant formula spray drying lines has the potential to reduce energy costs through higher yield and significantly enhance overall product quality.

## Author Contributions

**Ilyas Atalar:** conceptualization, methodology, formal analysis, writing – review and editing. **Nevzat Konar:** conceptualization, methodology, supervision, writing – review and editing. **Yamen Barakat:** conceptualization, methodology, formal analysis, writing – review and editing.

## Funding

This study was funded by Ankara University Scientific Research Projects Office (BAP), Project No: FBA‐2025‐4229.

## Conflicts of Interest

The authors declare no conflicts of interest.

## Data Availability

The data that support the findings of this study are available from the corresponding author upon reasonable request.
